# Data from roadside screening for psychoactive substances, alcohol and illicit drugs, among Spanish drivers in 2015

**DOI:** 10.1016/j.dib.2017.09.026

**Published:** 2017-09-20

**Authors:** Maria Jesús Herrero, Antonia Domingo-Salvany, Rafael de la Torre

**Affiliations:** a Reumathology Service, Parc de Salut Mar, Spain; b Drug Abuse Epidemiology Research Group, Institut Hospital del Mar d'Investigacions Mèdiques de Barcelona - IMIM, Doctor Aiguader 88, E-08003 Barcelona, Spain; c Integrative Pharmacology and Systems Neurosciences Research Group, Institut Hospital del Mar d’Investigacions Mèdiques - IMIM, Barcelona, Spain

## Abstract

The data presented in this article are related to the paper “Prevalence of psychoactive substances, alcohol and illicit drugs, in Spanish drivers: A roadside study in 2015”. (https://doi.org/10.1016/j.forsciint.2017.07.005) Domingo-(Salvany et al., 2017) [1]. In that paper it was not possible to directly compare 2015 results with previous editions for various reasons, one of which was the lack of a similar weighting procedure. The present paper provides 2015 figures of roadside screening tests which are weighted for traffic flow intensity and therefore allow direct comparisons with the screening tests conducted among Spanish drivers in 2008 and 2013.

**Specifications Table**

**Table:** 

Subject area	*Chemistry, Biology*
More specific subject area	*Roadside alcohol and illegal drug screening*
Type of data	*Table*
How data was acquired	*Survey among drivers*
Data format	*Data weighted by traffic flow intensity*
Experimental factors	
Experimental features	
Data source location	*Selected control sites in Spain*
Data accessibility	*In this article*

**Value of the data**

• The data provided allows comparison of screening test results with previous editions, as they have been weighted for traffic intensity at selected sites.
• The large sample size of this survey means that results can be considered stable, and as representativeness was pursued in the sampling procedure, the data can be used for comparisons.

## 1 Data

In this document we provide new data derived from 2015 roadside screening tests. Published results from previous editions of surveys conducted in Spain in 2008 and 2013 following the methodology set up in the framework of the European Union DRUID project (Driving Under the Influence of Drugs) [2] were weighted for traffic intensity. In the 2015 edition traffic intensity was measured in a slightly different way than previous ones and the results were published without weighting [1].

## 2 Materials and methods

Methodology for the three surveys has been already published. Detailed information about sample planning (considering both geographical and temporal aspects), driving recruitment, data collection and toxicological aspects can be found elsewhere [1], [3], [4]. Data obtained at the roadside with commercial screening instruments in 2015, after weighting, is presented. Alcohol concentration in exhaled air was measured in mg alcohol/liter by the Dräger Alcotest® (Alcotest 7110 MKIII), breath test being considered positive if the concentration was >0.05 mg/L. Measurements in oral fluid for other substances were done through the Dräger DrugTest® 5000 (Dräger Safety AG & Co, Lübeck, Germany) device. Cut-off concentrations applied were as follows: opiates (morphine), 20 ng/mL; amphetamines (D-amphetamine), 50 ng/mL; methamphetamine (D-methamphetamine), 35 ng/mL; cocaine (cocaine), 20 ng/mL; and THC (Delta-9-THC), 25 ng/mL. Weights have been applied considering traffic flow in terms of geographical area, urban/interurban environment and day of the week and time (day/night). This was done through information gathered at the roadside during driver recruitment. Every recruitment session was accompanied by traffic intensity information (number and type of vehicles at the selected point and specific timeframe).

## 3 **Results**

Results from the 2015 survey are presented with the same format as used in the 2008 and 2013 survey publications [3], [4] which were already reported after applying weights to balance for overall intensity of traffic flow.

Data is provided in Table 1.

**Table 1:** Proportion of drivers with positive results for alcohol and/or illegal drugs screened at the roadside. Spain, 2008, 2013 and 2015.

	DRUID-2008^a^*n*=3302		DRUID-2013^b^*n*=2932		EDAP-2015 *n*=2744	
	*n*	% (95% CI)	*n*	% (95% CI)	*n*	% (95% CI)
Tested negative (−) for alcohol or drugs	2763	83.7 (82.4–84.9)	2578	87.9 (86.7–89.1)	2406	87.7 (86.5–88.9)
Tested positive (+) for alcohol or drugs	539	16.3 (15.1–17.6)	354	12.1 (10.9–13.3)	339	12.3 (11.1–13.6)
+ alcohol/− drugs	151	4.6 (3.9–5.3)	97	3.30 (2.7–3.9)	33	1.2 (0.8–1.6)
− alcohol/+ drugs	321	9.7 (8.7–10.7)	233	8.0 (7.0–8.9)	293	10.7 (9.5–11.8)
+ alcohol/+ drugs	67	2.0 (1.5–2.5)	24	0.8 (0.5–1.1)	13	0.5 (0.2–0.7)

a [3].

b [4].
